# Killing two birds with a single stone—genetic manipulation of cytokinin oxidase/dehydrogenase (*CKX*) genes for enhancing crop productivity and amelioration of drought stress response

**DOI:** 10.3389/fgene.2022.941595

**Published:** 2022-07-18

**Authors:** Aman Sharma, Subasty Prakash, Debasis Chattopadhyay

**Affiliations:** National Institute of Plant Genome Research, New Delhi, India

**Keywords:** cytokinin oxidases/dehydrogenase, cytokinin homeostasis, root-shoot architecture, bio-fortification, sink strength, source capacity

## Abstract

The development of high-yielding, bio-fortified, stress-tolerant crop cultivars is the need of the hour in the wake of increasing global food insecurity, abrupt climate change, and continuous shrinking of resources and landmass suitable for agriculture. The cytokinin group of phytohormones positively regulates seed yield by simultaneous regulation of source capacity (leaf senescence) and sink strength (grain number and size). Cytokinins also regulate root-shoot architecture by promoting shoot growth and inhibiting root growth. Cytokinin oxidase/dehydrogenase (CKX) are the only enzymes that catalyze the irreversible degradation of active cytokinins and thus negatively regulate the endogenous cytokinin levels. Genetic manipulation of *CKX* genes is the key to improve seed yield and root-shoot architecture through direct manipulation of endogenous cytokinin levels. Downregulation of *CKX* genes expressed in sink tissues such as inflorescence meristem and developing seeds, through reverse genetics approaches such as RNAi and CRISPR/Cas9 resulted in increased yield marked by increased number and size of grains. On the other hand, root-specific expression of *CKX* genes resulted in decreased endogenous cytokinin levels in roots which in turn resulted in increased root growth indicated by increased root branching, root biomass, and root-shoot biomass ratio. Enhanced root growth provided enhanced tolerance to drought stress and improved micronutrient uptake efficiency. In this review, we have emphasized the role of *CKX* as a genetic factor determining yield, micronutrient uptake efficiency, and response to drought stress. We have summarised the efforts made to increase crop productivity and drought stress tolerance in different crop species through genetic manipulation of *CKX* family genes.

## 1 Introduction

Demand for staple food crops is expected to increase by up to 60% as the global human population is expected to reach the 9.6 billion mark by 2050 (Zhu et al., 2020). According to the latest estimates, around 928 million people were severely food insecure and 768 million people were undernourished in 2020 (FAO, 2021). The breeding programs have primarily focused on increasing crop yield and productivity but an equally important objective in the breeding programs is the improvement of micronutrient composition and density which has been largely overlooked to date (Gao et al., 2019). Increased risk of climate change has escalated the adverse effects of abiotic stresses on crop production. Heat and drought stress has proved to be the two most prominent constraints on the growth and production of crop plants (Fahad et al., 2017). Drought conditions cause yield losses up to 30%–90% depending on crop species, crop growth stage at the onset of drought, and type of harvested agriculture product (Dietz et al., 2021). As the rates of yield increase brought about by the ‘Green revolution’ have started to decline, there is an immediate and urgent need to increase crop productivity both in terms of quality and quantity by developing robust high yielding, bio-fortified and, stress-tolerant crop cultivars to meet growing food demands in the face of abrupt climate change, increased risk of biotic and abiotic stress and decreased availability of resources and landmass suitable for agriculture (Chen et al., 2020a; Zhu et al., 2020).

The ‘Green Revolution’ was focused on the signaling associated with phytohormone gibberellins. It was reported that the semi-dwarf cereal varieties compromised in either gibberellin biosynthesis (rice) or gibberellin response (wheat and maize) had semi-dwarf phenotypes, less prone to lodging, and high seed yield. The reason behind the higher seed yield of these varieties was the reallocation of a greater proportion of photoassimilates to the reproductive tissues instead of vegetative tissues (Chen et al., 2020a; Liu et al., 2020a). Moreover, the cultivation of these varieties is resource-intensive, requiring huge inputs of fertilizers, water, and pesticides (Chen et al., 2020a; Jameson and Song, 2020a). Yield enhancement brought about by the ‘Green Revolution’ has started to flatten due to climate change and resource limitations (Zhu et al., 2020). Cytokinins are poised to underpin the second “Green Revolution” because of their recognized effects on increasing seed yield and overall plant architecture. Additionally, the possibility to alter the endogenous cytokinin levels through genetic manipulation of genes associated with cytokinin homeostasis might be useful in minimizing the effects of various environmental stresses and nutrient deficiencies (Jameson and Song, 2020a). The cytokinin group of phytohormones is involved in the regulation of multiple aspects of plant growth and development (Kieber and Schaller, 2018a), many of which have direct implications for crop improvement, such as the regulation of root-shoot architecture (Jing and Strader, 2019; Kurepa and Smalle, 2022), regulation of inflorescence meristem activity and seed yield output (Ashikari et al., 2005; Bartrina et al., 2011a; Schwarz et al., 2020a), regulation of leaf senescence and photosynthesis (Hönig et al., 2018), nutrient uptake (Gao et al., 2019; Kurepa and Smalle, 2022) and responses to biotic and abiotic stresses (Cortleven et al., 2019; Liu et al., 2020b; Li et al., 2021b; Salvi et al., 2021). Thus, there are clear implications of endogenous cytokinin level manipulation in altering plant architecture for the development of robust stress-tolerant crop varieties having optimum root-shoot architecture along with high yield output (Jameson and Song, 2020a). Cytokinin oxidase/dehydrogenase (CKX) is the only enzyme of cytokinin homeostasis maintenance that catalyzes the irreversible degradation of active cytokinins and thus, is an important negative regulator of endogenous cytokinin concentration. Genetic manipulation of genes encoding cytokinin oxidase/dehydrogenase is the key to spatiotemporal regulation of endogenous cytokinin levels. This review aims to summarise the efforts made to establish *CKX* as a genetic target for yield improvement, enhanced bio-mineral accumulation, and drought stress tolerance. Starting with a brief introduction to cytokinin biosynthesis and homeostasis we further describe the structure, function, and evolution of cytokinin oxidases/dehydrogenases enzymes along with a brief review of the *CKX* gene family in flowering plants. Next, the role of *CKX* as a genetic target for yield improvement, root-shoot architecture improvement, drought stress tolerance, and enhanced micronutrient acquisition is discussed in detail followed by a conclusion and future directions.

### 1.1 Cytokinin homeostasis: A regulatory web to regulate local cytokinin concentration

**FIGURE 1:**
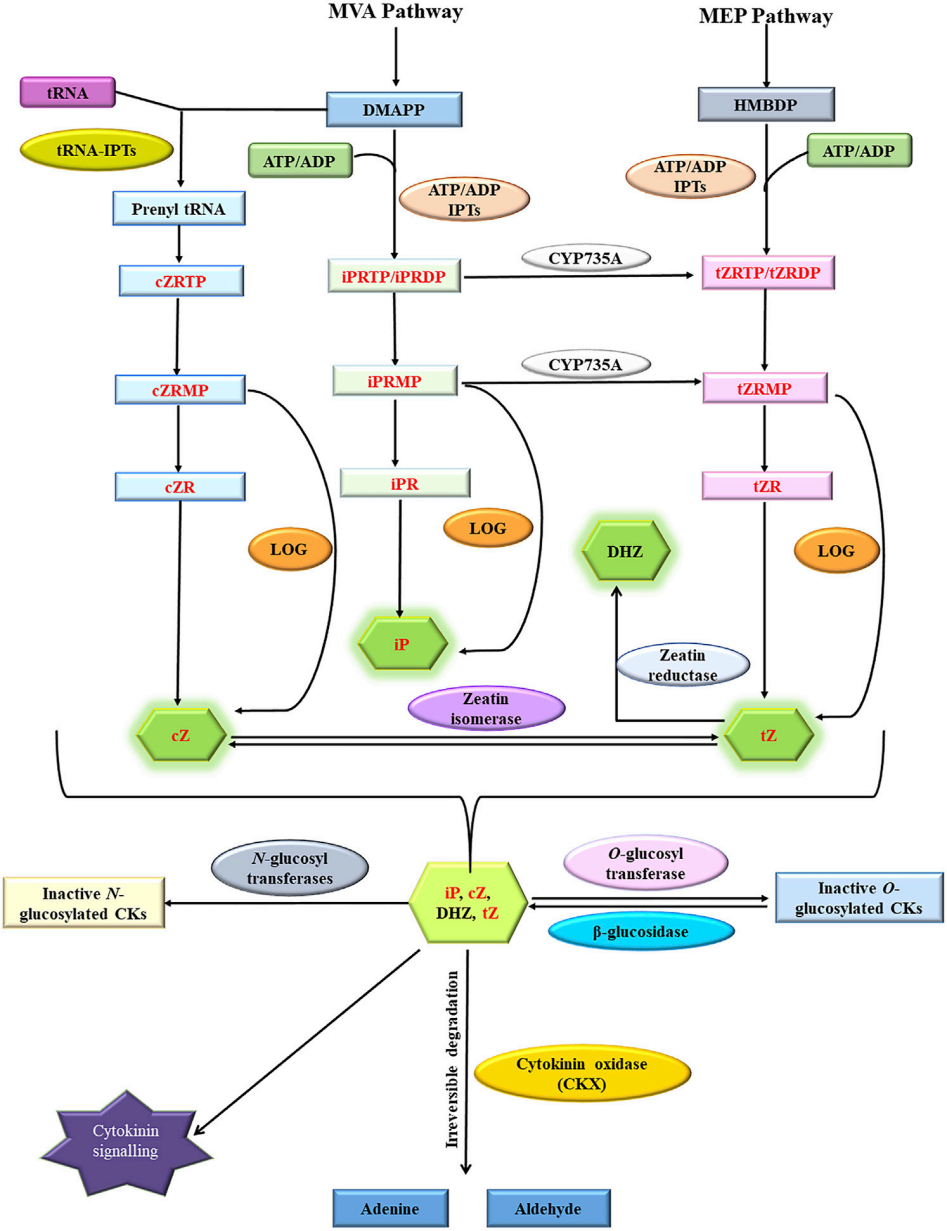
Cytokinin homeostasis maintenance pathway involving *in situ* cytokinin biosynthesis (catalyzed by ATP-ADP IPTs and tRNA-IPTs), activation (catalyzed by specific phosphoribohydrolases encoded by *LOG* genes), irreversible inactivation (catalyzed by *N*-glucosyltransferases), reversible inactivation (catalyzed by *O*-glucosyltransferases), reactivation (catalyzed by *β*-glucosidases) and irreversible degradation (catalyzed by cytokinin oxidase/dehydrogenases). Cytokinin oxidase/dehydrogenases substrates are highlighted in red (Modified from Frébort et al., 2011b; Kieber and Schaller, 2014; Hluska et al., 2021b).

Cytokinins are N⁶-substituted adenine-derived phytohormones having either isoprene or aromatic side chain as N⁶ substitution (Kieber and Schaller, 2018a). Dihydrozeatin (DHZ), Isopentenyl adenine (iP), *cis*-zeatin (cZ), and *trans*-zeatin (tZ) are the most common forms of cytokinins present in plants. Isopentenyl adenine transferases encoded by *ADP/ATP IPT* genes catalyze the transfer of the isoprenyl side chain from Dimethyl allyl diphosphate (DMAPP) and (E)-4-hydroxy-3-methylbut-2-enyl diphosphate (HMBDP) to adenine ring of either ATP or ADP resulting in the synthesis of isopentenyl adenine ribonucleotide triphosphate/diphosphate (iPRTP/iPRDP) and trans-zeatin ribonucleotide triphosphate/diphosphate (tZRTP/tZRDP) respectively which are further converted into respective monophosphates (iPRMP and tZRMP) (Miyawaki et al., 2006; Frébort et al., 2011a). iPRTP/iPRDP/iPRMP can also be converted into respective trans-Zeatin counterparts by cytokinin trans-hydroxylases encoded by *CYP735A* genes (Takei et al., 2004). Enzymes encoded by *t-RNA IPT* genes catalyze the addition of isoprenyl side chain to adenine ring in specific tRNA molecules and are thought to be responsible for the synthesis of cis-Zeatin (cZ) type of cytokinins (Miyawaki et al., 2006; Hluska et al., 2021a). Cytokinin ribotides (iPRMP/tZRMP/cZRMP) are converted into bioactive free base forms (iP, tZ, and cZ) by specific phosphoribohydrolases encoded by *LONELY GUY (LOG)* genes (Kurakawa et al., 2007; Kuroha et al., 2009; Frébort et al., 2011a). Sugars can be attached to hydroxyl side chains of cZ, tZ, and DHZ by *O*-glucosyltransferase (*ZOG*) resulting in reversible inactivation (Martin et al., 1999; Veach et al., 2003). The resulting sugar conjugates can be again converted back into active forms by β-glucosidases encoded by *GLU* genes (Brzobohatý et al., 1993; Kieber and Schaller, 2014). The addition of sugars to N⁷ and N⁹ position of the adenine ring of active cytokinins by N-glucosyltransferases renders them permanently inactive (Šmehilová et al., 2016). Cytokinin oxidase/dehydrogenase encoded by *CKX* genes facilitates the irreversible degradation of both free base and nucleotide forms of cytokinins (Figure 1) (Kieber and Schaller, 2014; 2018b). Many genes involved in cytokinin homeostasis maintenance such as *IPT* (Wang et al., 2020a)*, LOG*(Chen et al., 2022)*, CKX* (Chen et al., 2020b)*,* glucosyltransferases (*ZOG*) (Chen et al., 2021)*,* and cytokinin trans-hydroxylase (*CYP735A*) (Takei et al., 2004) are present as small multigene families in plants. Different members often exhibit distinct spatio-temporal expression patterns and encode gene products having distinct substrate specificity, subcellular localization, and physico-chemical activities facilitating very fine spatio-temporal regulation of different cytokinins metabolites.

### 1.2 Cytokinin oxidases/dehydrogenases—structure, function, and evolution

Cytokinin oxidase/dehydrogenases (CKX, EC 1.5.99.12) are the flavoprotein oxidoreductase enzymes capable of catalyzing the irreversible cytokinin degradation by cleaving the isoprenyl side chain attached to the N⁶ position of the adenine ring (Avalbaev et al., 2012). Cytokinin oxidases/dehydrogenases degrade freebase active forms such as iP, tZ, cZ, and their respective nucleoside and nucleotide forms by catalyzing the secondary amine group and the resulting imine is non-enzymatically hydrolyzed into adenine or adenosine and corresponding aldehydes (Frébort et al., 2011b). DHZ and cytokinins having aromatic side chains such as benzylaminopurine (BAP) and kinetin are not degraded by CKX. Crystal structures of ZmCKX1 (ZmCKO1), ZmCKX2 (ZmCKO2), ZmCKX3 (ZmCKXO3), and ZmCKX4 (ZmCKO4) from maize and AtCKX7 from *Arabidopsis thaliana* has been resolved (Malito et al., 2004; Bae et al., 2008; Kopečný et al., 2016). Structures of both maize and *Arabidopsis* CKX exhibit a two-domain topology of vanillyl oxidase family proteins consisting of an N-terminal FAD-binding domain and C-terminal substrate binding domain (Malito et al., 2004; Bae et al., 2008; Gouda et al., 2020; Frébortová and Frébort, 2021). In ZmCKX1*,* the FAD-binding domain spans between amino acid residues 40–244 and 492–534 while residues 245–491 constitute the substrate binding domain (Malito et al., 2004; Kopečný et al., 2016). In AtCKX7, the FAD-binding domain is composed of residues 34–237 and 480–514 while residues 238–479 constitute the substrate binding domain (Bae et al., 2008). The active site of CKX enzymes is bipartite in structure consisting of a funnel-shaped region on the surface that binds adenine moiety of cytokinin substrates and an internal cavity situated above the isoalloxazine ring of the FAD cofactor serving as the binding site for the aliphatic side chain. Asp169-Glu288 pair is an important element in the active site which polarises the N10 atom of the side chain which in turn facilitates the transfer of two protons and one hydrogen atom from C11 of the side-chain to N5 of the FAD cofactor. The intermediate imine product is further hydrolyzed to yield reaction products. Reduced FADH₂ is re-oxidized by electron acceptors such as quinones (dehydrogenase mode) or molecular oxygen (oxidase mode). However, the existence of any tunnel or cavity serving as the binding site for electron acceptor in the vicinity of the FAD cofactor remains elusive (Malito et al., 2004; Kopečný et al., 2016). Amino acid residues pertaining to cofactor binding and substrate binding are conserved between maize and *Arabidopsis* CKXs, indicating that the catalytic mechanism is conserved between dicot and monocot plants (Bae et al., 2008). Cytokinin oxidases/dehydrogenases are encoded by small multigene families in higher plants and different members of gene families often exhibit distinct spatio-temporal expression patterns and their gene products exhibit different subcellular localization, substrate specificities, and physic-chemical properties (Joshi et al., 2018; Chen et al., 2020a; Gouda et al., 2020). There are 7 *CKX* homologs in the model dicot plant *A. thaliana* (Schmülling et al., 2003), 11 in rice (Ashikari et al., 2005; Rong et al., 2022), 13 in maize (Zalabák et al., 2014), 11 in barley (Gasparis et al., 2019), 11–14 in bread wheat (Chen et al., 2020a), 10 in chickpea (Khandal et al., 2020), 17 in soybean (Nguyen et al., 2021a), 9 in model legume *Medicago truncatula* (Wang et al., 2021a), 23 in *Brassica napus* (Liu et al., 2018; Schwarz et al., 2020a)*,* 34 in *Brassica olearacea* (Zhu et al., 2022), 11 in C₄ model plant *Setaria italica* (Wang et al., 2014a)*, 8* in *Vitis vinifera* (Yu et al., 2021) and 7 in biodiesel plant *Jatropha curcas* (Cai et al., 2018) (Table 1).

**TABLE 1:** Features of *CKX* gene family members in selected plant species.

S.No	Plant species	Number of *CKX* genes	Range of ORF size/gene size (base pairs)	Range of encoded polypeptide length (number of amino acids)	Range of molecular weight of encoded polypeptides (KDa)	spatio-temporal expression pattern	References
Model plant							
1	*Arabidopsis thaliana*	7	1,506–1728	501–575	—	Inflorescence meristem and floral meristem: *AtCKX3* and *AtCKX5* Vascular tissue, transmitting tissue, and embryo sac: *AtCKX7*	Schmülling et al. (2003); Bartrina et al. (2011a); Köllmer et al. (2014)
Cereal crops							
2	*Oryza sativa*	11	1,506–1863	501–620	—	*Inflorescence meristem*: *OsCKX2* Senescing, leaves and developing grains: *OsCKX11,* Leaf-blade, roots, and shoot base: *OsCKX4*	Ashikari et al. (2005); Gao et al. (2014); Zhang et al. (2021); Rong et al. (2022a); Mameaux et al. (2012)
3	*Hordeum vulgare*	At least 11	699–1842	233–614	—	14 *DAP spikes*: HvCKX1, HvCKX9, HvCKX4, and HvCKX11, Leaves: HvCKX9, HvCKX5, and HvCKX11	Zalewski et al. (2014)
4	*Zea mays*	13	804–2066	267–568	—	*Constitutive expression*: *ZmCKX6, ZmCKX10,* and *ZmCKX1,* Young and mature leaves: *ZmCKX2* and *ZmCKX3, Reproductive organs: ZmCKX4* and *ZmCKX4b*	Mameaux et al. (2012); Gu et al. (2010)
Millets							
5	*Setaria italica*	11	720–1,620	239–539	—	Expression of all the *SiCKX* genes induced in germinating embryo when treated with 6-BAP. Induced by salinity and drought treatment: SiCKX1, SiCKX3, SiCKX5, and SiCKX8	Wang et al. (2014b)
Legume crops							
6	*Medicago truncatula*	9	1,530–1,644	509–547	56.8–62.2	Flowers; Medtr7g090920 and Medtr4g126150/MtCKX2. Leaves: Medtr4g044110. Roots and root nodules: Medtr4g126160	Wang et al. (2021a)
Oilseed crops							
7	*Brassica napus*	23	1,011–2,307	336–768	33–87	Systematic/constitutive: *BnCKX5-1, BnCKX5-2* and, *BnCKX7-3.* Silique pericarp: *BnCKX3-2* and *3–3. Buds: BnCKX3-1*. Inflorescence meristem, floral meristem, and developing gynoecia: *BnCKX3* and *BnCKX5*	Liu et al. (2018); Schwarz et al. (2020a)
8	*Glycine max*	17	1,329–1,659	442–552	49–62	Reproductive organs: *GmCKX7-1, GmCKX1-1, GmCKX1-2, GmCKX5-2, GmCKX7-3* and, *GmCKX3-1.* Seeds: *GmCKX7-1*	Nguyen et al. (2021a)
Cash crops							
9	*Jatropha*	7	1,332–1,650	443–549	—	Roots: *JcCKX1* and *JcCKX7.* Female flowers: *JcCKX1, JcCKX2* and *JcCKX4.* Male flowers: *JcCKX3.* Seeds: *JcCKX4* and *JcCKX2.* Leaves: *JcCKX4* and *JcCKX5.* All tissues: *JcCKX6*	Cai et al. (2018)
Fruits crops							
10	*Vitis vinifera*	8	1,279–1961	424–632	47–70	Inflorescence: VvCKX4 and VvCKX8	Yu et al. (2021)
11	*Malus domestica*	12	942–2,535	313–844	34–96	Leaves: MdCKX4, MdCKX5 and, MdCKX8. Root: MdCKX1, MdCKX7, MdCKX9 and, MdCKX11/12	Tan et al. (2018)

There is an apparent functional dichotomy in *CKX* gene family members in flowering plants. Recent reports propose that two categories of *CKX* genes exist in flowering plants, which are ancient and non-ancient *CKXs*. The differences between these two groups have been reported in terms of their subcellular localization, biochemical properties, roles in growth and development, and respective substrate specificities. Ancient *CKX* genes are confined to 1–2 gene copies in each species and their encoded proteins are believed to play housekeeping roles, preferentially degrade cZ, exhibit nearly uniform expression across tissues, and are localized to cytosol while non-ancient *CKX* genes are present in variable numbers across different species and the proteins encoded by them are involved in the regulation of organ development and stress responses, preferentially degrade iP and tZ type of cytokinins, exhibit differential spatio-temporal expression induced rapidly under environmental stresses and are found to be present in cellular compartments such as ER, vacuole and apoplast. *AtCKX7, ZmCKX10,* and *OsCKX11* are the representatives of ancient *CKXs* in *Arabidopsis*, maize, and rice respectively (Wang et al., 2020b). Interestingly similar functional dichotomy was also observed among *IPT* gene family members (Wang et al., 2020c). A recent report indicates that this apparent duality is a characteristic feature of cytokinin metabolism (Hluska et al., 2021b).

*CKX* genes are present in angiosperms, non-vascular and vascular seeded plants, some groups of bacteria, and a few fungi species. Surprisingly, they are altogether absent in algae. Although homologs of cytokinin biosynthesis genes (*IPT* and *LOG*) were reported in a few archaeal species, there is no information available about *CKX* homologs in any archaeal species (Wang et al., 2020a; Frébortová and Frébort, 2021). Initial reports hypothesized that plants acquired *CKX* genes by lateral transfer from cyanobacteria through chloroplast (Frébort et al., 2011b). Recent reports suggest the transfer of *CKX* genes into the plants from an ancient chlamydial endosymbiont ancestor that resided inside the common ancestor of plants (Wang et al., 2020b). Another group hypothesized that bacterial and plant *CKXs* might have originated independently from ancient and omnipresent FAD-linked oxidases. Their model indicates that algae might have derived FAD-linked oxidases from either the proteobacteria (modern mitochondria) or cyanobacteria (modern chloroplast) or even directly from the most common ancestor of eukaryotes. *CKX* genes present in proto-mitochondria and proto-chloroplast were lost during algal evolution, explaining the absence of *CKX* homologs in algae. FAD-linked oxidase genes derived from mitochondria or chloroplast or common ancestors later on duplicated, diverged, and expanded to give rise to modern plant *CKX* gene families, independent of bacterial *CKX* homologs (Dabravolski and Isayenkov, 2021).

### 1.3 Cytokinin oxidases as a genetic target for crop yield improvement

**FIGURE 2:**
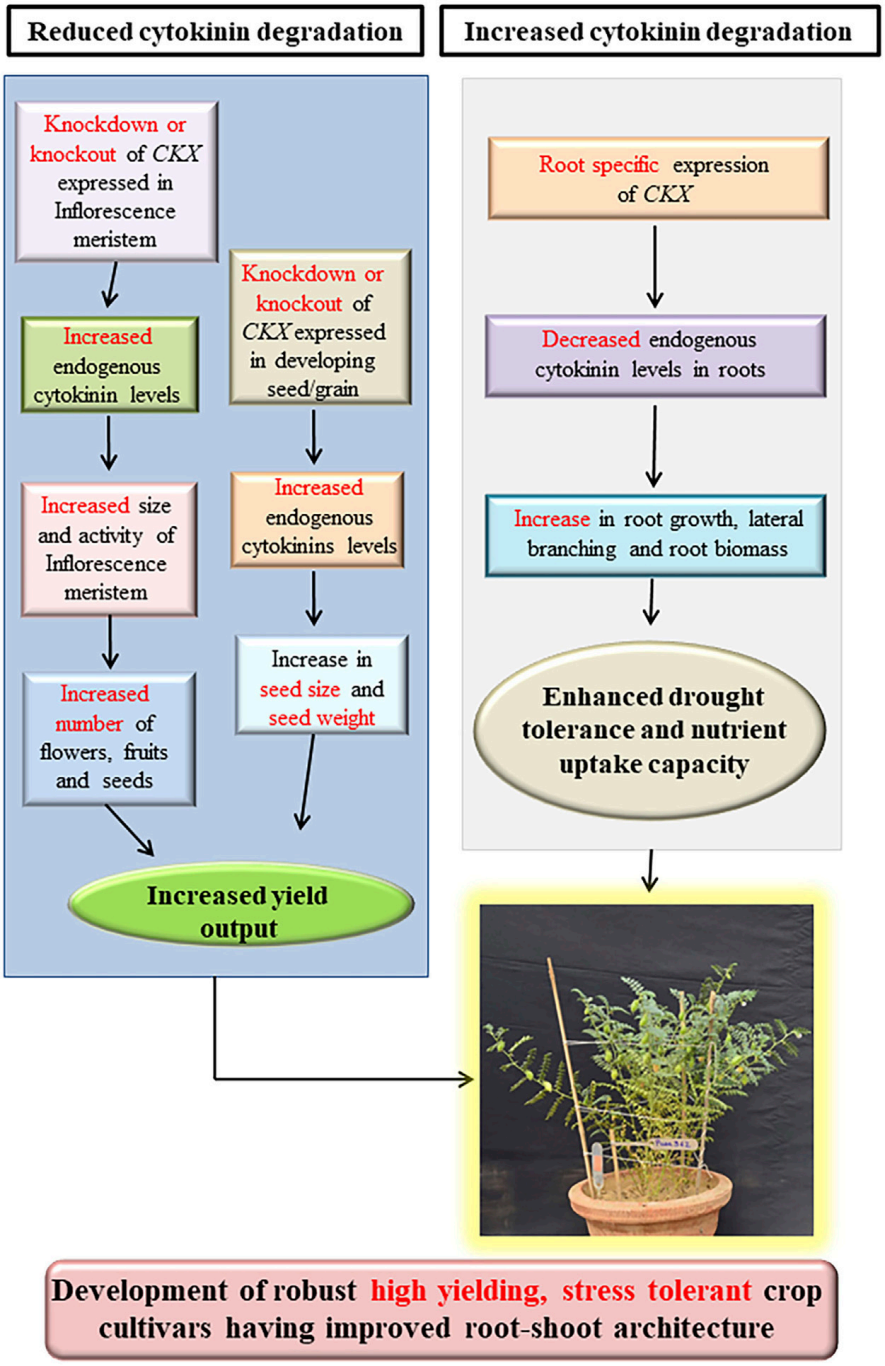
Combined strategy for the development of high yielding, biofortified, drought-tolerant crop cultivars through alteration of endogenous cytokinin by genetic manipulation of cytokinin oxidase/dehydrogenase family genes (Modified from (Jameson and Song, 2020b).

Cytokinins positively regulate both source capacity (photosynthetic efficiency) and sink strength (number of ovules formed in gynoecia as well as the number and weight of seeds) by directly regulating leaf senescence and inflorescence meristem activity and thus they are key regulators of yield output (Schwarz et al., 2020a). Endogenous cytokinin levels of different crops might be manipulated by different approaches based on whether a crop is source limited or sink limited, to increase the yield output (Jameson and Song, 2016a). Besides regulating inflorescence meristem activity, cytokinins are also indispensable for seed development indicated by a sharp increase in endogenous cytokinin levels in cereals crops such as maize, wheat, barley, and rice shortly after anthesis (Wheeler, 1972a; Yang et al., 2000, 2003; Chen et al., 2020a). These rising endogenous cytokinin levels coincide with the increased cell division rates and cell number in developing grain endosperm during grain filling stages resulting in the formation of a strong “Sink” which eventually enhances photoassimilate migration and accumulation during grain development (Wheeler, 1972b; Yang et al., 2000, 2003; Jameson and Song, 2016a). Cytokinins positively regulate inflorescence meristem activity and seed yield (Bartrina et al., 2011b; Jameson and Song, 2016b). As CKXs catalyze the irreversible degradation of cytokinins and thus negatively regulate inflorescence and floral meristem activity (Bartrina et al., 2011b; Schwarz et al., 2020b). Genetic manipulation of *CKX* genes (knockdown and knockout) expressed in reproductive organs and developing seeds provides us with an opportunity for increasing seed yield through endogenous cytokinin level alteration. Thus, *CKX* becomes an ideal target for yield improvement (Chen et al., 2020b) (Figure 2). Several studies have established the role of *CKX* as a major regulator of seed yield as reviewed below (Table 2).

**TABLE 2:** Genetic manipulation of *CKX* genes in different plant species for increasing seed yield output.

S.No.	Name of *CKX* gene	Source species	Target species	Type of genetic manipulation	Observed changes in yield-related parameters	Comments	References
1	*OsCKX2*	*Oryza sativa*	*Oryza sativa*	Natural genetic variation	Panicle branches ↑		Ashikari et al. (2005)
					grain number per panicle ↑		
					Total grain number ↑		
				RNAi mediated gene knockdown	Number of tillers ↑	Delayed Leaf senescence (DLS) phenotype in transgenic lines	Yeh et al. (2015)
					Number of panicles ↑		
					Grains per plant ↑		
					TGW ↑		
					Total grain yield per plant ↑		
					Plant height ↑	Reduced yield loss in transgenic lines under salinity stress compared to control lines	Joshi et al. (2018)
					Filled grains per panicle ↑		
					Panicle number ↑		
					Harvest index ↑		
					TGW ↑		
					Leaf senescence ↑		
				CRISPR/Cas9 mediated gene knockout	Plant height ↑	Effect on seed yield output not reported	Li et al. (2016)
					Panicle length ↑		
					Flower number per panicle↑		
				EMS mutagenesis is followed by Map-based cloning	Panicle length ↑		Tsago et al. (2020)
					Panicle branches ↑		
					Filled grains per plant ↑		
					Grain length ↑		
					TGW ↑		
					Seed setting rate ↑		
2	*OsCKX11*	*Oryza sativa*	*Oryza sativa*	CRISPR/Cas9 mediated gene knockout	Tiller’s number ↑	*OsCKX11* is involved in the regulation of both leaf senescence and seed yield	Zhang et al. (2021)
					Grains per panicle ↑		
					Grains per plant ↑		
					Fertility rate ↓		
					TGW ↓		
					Leaf senescence ↓		
3	*TaCKX1*	*Triticum aestivum*	*Triticum aestivum*	RNAi mediated gene knockdown	Number of spikes ↑		Li et al. (2018)
					Grain yield ↑		
					TGW ↓		
4	*HvCKX1*	*Hordeum vulgare*	*Hordeum vulgare*	RNAi mediated gene knockdown	Spikes number ↑		Holubová et al. (2018)
					Grains per plant ↑		
					Overall grain yield ↑		
					TGW ↓		
5	*HvCKX1 and HvCKX3*	*Hordeum vulgare*	*Hordeum vulgare*	CRISPR/Cas9 mediated gene knockout	In one *ckx3* mutant line	Activation of strong cytokinin homeostatic response resulted in reduced expression of CK biosynthetic genes and increased CK inactivation through *O*-glucosylation	Gasparis et al. (2019)
					Spikes number ↑		
					In other mutant lines		
					Grain number ↓		
					Grain weight ↓		
6	*CKX3/CKX5*	*Arabidopsis thaliana*	*Arabidopsis thaliana*	*ckx3ckx5* double mutant generated by random T-DNA mutagenesis	Flowers number per plant ↑	*ckx3* or *ckx5* mutations alone were not sufficient to alter yield components significantly	Bartrina et al. (2011a)
					Flower size ↑		
					Gynoecium size ↑		
					Inflorescence meristem size ↑		
					Ovules per gynoecium ↑		
					Siliques per plant ↑		
					Seed number per silique ↑		
					Overall seed yield ↑		
		*Brassica napus*	*Brassica napus*	*ckx3ckx5 sextuple* mutant generated by random T-DNA mutagenesis and TILLING.	Inflorescence meristem size ↑	The number of seeds per silique was equivalent to wild type because of high seed mortality	Schwarz et al. (2020)
					Floral primordia number ↑		
					Flower number per plant ↑		
					Gynoecium size ↑		
					Ovules per gynoecium ↑		
					Seed weight and number ↑		
					TGW ↑		
7	*MtCKX3*	*Medicago truncatula*	*Medicago truncatula*	*Tnt1* retrotransposon tagged insertion mutants	Length of primary root ↑	Single mutants of any other *CKX* gene did not exhibit significant deviation from wild-type phenotype, indicating functional redundancy	Wang et al. (2021)
					Lateral roots number ↓		

^#^’↑’ and ‘↓’ symbols respectively indicate increase and decrease in corresponding parameter.

#### 1.3.1 Rice

Rice grain number locus *Gn1a* is encoded by *OsCKX2*and it was found to be responsible for higher yield i.e., increased panicle branching, increased grain number per panicle as well as per plant in two high yielding rice varieties Habataki and 5150. There was reduced expression of *OsCKX2* in Habataki inflorescence meristem as compared to the Koshikari variety while in the case of 5150 there was no expression of *OsCKX2* in inflorescence meristem. Sequence comparison between Koshikari and Habataki alleles indicated that the Habataki allele had a 16bp deletion in the 5′-UTR, a 6bp deletion in the first exon, and 3 nucleotide changes causing amino acid sequence variation in exons 1 and 4 while the 5150 allele had 11 bp deletion in the coding region resulting in the generation of a premature stop codon. Among these 3 varieties, 5150 exhibited the highest yield followed by Habataki and Koshikari, indicating that *OsCKX2* negatively regulates yield output in rice (Ashikari et al., 2005). Downregulation of *OsCKX2* using RNAi resulted in delayed leaf senescence, increased tiller number (15%–43%), increased panicle number (27%–81%), increased grain number per plant (44%–67%), increased total grain weight per plant (58%–75%) and increased thousand-grain weight (TGW) (5%–15%) in homozygous T₃ transgenic lines (Yeh et al., 2015). Gene knockout of *OsCKX2* using CRISPR/Cas9 resulted in a nearly two-fold increase in the number of flowers per plant as well as increased plant height and panicle length in T₂ homozygous lines having frameshift mutations. Although the effect on yield output *per se* was not reported (Li et al., 2016). Transgenic lines developed through downregulation of *OsCKX2* utilizing RNAi exhibited increased yield output and reduced yield penalty under salinity stress. Homozygous T₂ transgenic lines showed significantly reduced senescence, increased panicle branching, increased panicle number per plant (50%), increased TGW (10%), increased number of filled grains per panicle (69.7%), and higher harvest index (HI) (25.4%) as compared to the non-transgenic control lines under normal conditions while under salinity stress conditions transgenic lines had increased panicle number (80%), increased TGW (26%), increased number of filled grains per panicle (196.4%), and increased HI (136.4%) compared to non-transgenic control lines. Transgenic lines retained higher chlorophyll and carotenoids content, relative water content (RWC), net photosynthetic rate (NPR), stomatal conductance, intercellular CO₂ concentration, electron transport rate (ETR), and maximal photosystem Ⅱ efficiency (Fv/Fm ratio) relative to wild type control lines under salinity stress conditions which might have contributed to reduced yield penalty under salinity stress (Joshi et al., 2018). A mutant *OsCKX2-2* identified in rice through random mutagenesis and map-based cloning had an increased number of panicle branches and increased grain length, grain weight, TGW, and yield per plant compared to their wild-type counterparts (Tsago et al., 2020). Recently *OsCKX11* gene-knockout lines generated through CRISPR/Cas9 exhibited significantly delayed leaf senescence along with an increased number of tillers (14.83%–27.07%), increased grains per panicle (15.11%–27.96%), and increased grains per plant (21.62%–27.29%), while fertility and TGW decreased as compared to wild type plants. Small-scale field tests based on two independent plots showed that grain yield increased significantly (by 7.47% and 7.58%) in *osckx11* mutant lines compared to WT control lines. Leaves of osckx*11* knockout lines retained higher cytokinin levels, chlorophyll content, maximal photosystem Ⅱ efficiency (Fv/Fm), and accumulated lower ABA levels compared to control lines during the onset of senescence stage. Expression of ABA biosynthetic genes, senescence-associated genes, and chlorophyll catabolic genes was reduced while expression of ABA degradation genes was elevated in senescing leaves of knockout lines compared to control plants. OsCKX11 might positively regulate leaf senescence by modulating the expression of ABA, cytokinin, and chlorophyll metabolism genes at the transcription level. The results established the dual role of *OsCKX11* in the regulation of both leaf senescence (source capacity) and the number of grains (sink strength) simultaneously (Zhang et al., 2021).

#### 1.3.2 Barley

RNAi mediated gene silencing of *HvCKX1* which is predominantly expressed in spikes and roots lead to increased grain production (154–168 grains in transgenic lines compared to 117 in control lines), increased TGW (32 g in transgenic lines compared to 26 g in control lines), increased average yield per plant (5–5.5 g in transgenic lines as compared to 3.35 g in controls) as well as increased root mass (28.4–31.2 mg in transgenic lines as compared to 23.1 mg in control lines) in T₀ barley transgenic lines (Zalewski et al., 2010). Silencing of *HvCKX2* led to an increase in grain number to 144%–163% and total grain yield was 135%–167% higher as compared to control plants in T₀ barley transgenic lines. Homozygous T₁ transgenic lines also exhibited increased productivity marked by increased TGW, increased plant height (1–4.5 cm higher than control), and an increased number of spikes (0.5-2 spikes higher per plant as compared to control lines) (Zalewski et al., 2012). *HvCKX1* knockdown and knockout lines were generated by short hairpin RNAi and CRISPR/Cas9 respectively. Gene knockdown lines exhibited increased plant productivity under controlled conditions marked by the production of 10% more spikes, 40% more grains per plant, and overall yield up to 120% of the control plants (Holubová et al., 2018). Two knockdown lines grown under field conditions exhibited increased yield per unit area up to 120%–139% in 2016 and 118%–136% in 2017 as compared to control lines. However, the yield parameters of knockout lines were not reported (Holubová et al., 2018). Recently *HvCKX1* and *HvCKX3* knockout lines generated through CRISPR/Cas9 exhibited inconsistent, ambiguous, and unexpected phenotypes in terms of yield, all but one *ckx3* line exhibited a significantly lower number of spikes and grains as well as lower TGW while *ckx1* lines were equivalent to control lines in terms of yield parameters. Gene expression analysis and RNA seq data indicated that expression of cytokinin biosynthesis (*IPT*), activation (*LOG*) and, reactivation (*GLU*) genes were decreased while expression of cytokinin inactivating genes (*ZOG*) was increased in *HvCKX1* and *HvCKX3* knockout lines compared to control lines. Thus activation of strong cytokinin homeostasis maintenance response nullified the effect of *HvCKX1* and *HvCKX3* knockout (Gasparis et al., 2019b). RNAi-mediated gene knockdown might not have triggered the homeostasis response because contrary to gene knockout, gene knockdown does not result in complete abolishment of gene function (Gasparis et al., 2019c). *HvCKX3* was found to be involved in the maintenance of inflorescence architecture in barley under high-temperature stress. Barley MADS-box protein HvMADS1 binds to A-tract-rich CArG-box motifs in the promoter of the *HvCKX3* gene with high affinity at higher temperatures and activates its expression to regulate local cytokinin levels. Enhanced expression of *HvCKX3* under high-temperature results in reduced cytokinin response which causes repression of meristematic cell divisions, ultimately stabilizing meristem determinacy and facilitating the development and maintenance of unbranched spike architecture at elevated temperatures (Li et al., 2021a).

#### 1.3.3 Wheat

In wheat, *TaCKX6-D1* (recently renamed as *TaCKX2.2.1-3D*) situated on chromosome 3D was found to be an orthologue of *OsCKX2*. An 18bp deletion in the second intron of *TaCKX6-D1* was found to be associated with higher TGW based on an association analysis across 115 diverse cultivars. Varieties having haplotype *TaCKX6-D1b* (wild type *TaCKX6-D1* allele) exhibited 1.4-5.4fold higher expression as compared to those having haplotype *TaCKX6-D1a* (*TaCKX6-D1* allele having 18bp deletion in 2nd intron near 3′ splice site). Differences in promoter diversity such as methylation state between *TaCKX6-D1b* and *TaCKX6-D1a*allele, change in alternative splicing pattern caused due to 18 bp deletion or an upstream gene or cis-factor might be causing the observed difference in expression between *TaCKX6-D1b* and *TaCKX6-D1a* allele as no other polymorphism were detected between *TaCKX6-D1b TaCKX6-D1a* alleles. Although the exact molecular basis is still not known. Association analysis indicated that the mean TGW of varieties having haplotype *a* was 3.94 g higher than type b. Thus there was a significant negative correlation between *TaCKX6-D1* expression and TGW (Zhang et al., 2012). Allelic variation in *TaCKX6a02* (renamed as *TaCKX2.1-3D*) located on chromosome 3D was found to be significantly correlated with grain size, grain weight, and grain filling rate (GFR) based on the analysis of grain traits of the recombinant inbred lines (RILs) generated by crossing high yielding and low yielding wheat varieties (Lu et al., 2015a). The *TaCKX6a02a* allele having 29 bp insertion in 3′ UTR was found to be significantly associated with increased grain yield as a result of association analysis across 102 cultivars (Lu et al., 2015b). Although UTRs mediated regulation of gene expression is a very well-established phenomenon (Mayr, 2018; Srivastava et al., 2018), the exact molecular basis behind the association of 29 bp insertion in 3′UTR of *TaCKX6a02a* allele is not known (Lu et al., 2015a). *TaCKX2.4* gene knockdown through shRNA mediated RNAi resulted in a 5.8%–12.6% increase in the number of grains per spike in T₃ homozygous transgenic lines as compared to wild type control lines while the number of spikes, number of spikelets per spike, kernel length and width as well as TGW were identical to control lines (Li et al., 2018). Expression of *TaCKX2.4* was drastically reduced in the spikes of transgenic lines as compared to the non-transgenic lines indicating a negative correlation between *TaCKX2.4* expression and the number of grains per spike (Li et al., 2018). RNAi mediated strong gene silencing of *TaCKX1*; an orthologue of *HvCKX1* increased several yield parameters in T₂ transgenic lines including a higher number of spikes, higher number of grains, increased grain yield, and seedling root weight. On the other hand, plant height, length of the spike, and TGW were decreased as compared to control lines. Knockdown of *TaCKX1* resulted in simultaneous strong down-regulation of *TaCKX11* and *TaCKX1* and up-regulation of *TaCKX2.2, TaCKX5,* and *TaCKX9* in T₂ homozygous lines resulting in overall CKX activity being identical to control lines because of the cumulative contribution of isozymes encoded by different *CKX* genes. Still, cytokinin concentrations were increased by 23%–76% in transgenic lines as compared to control lines. As different CKX enzymes have different substrate preferences, the composition of the active cytokinin pool and phenotypic traits of the modified plants were changed accordingly (Jabłoński et al., 2020).

#### 1.3.4 Dicotyledonous plant species

Cytokinin status and its regulation by CKX is a very well-established determinant of yield in crops such as rice, barley, and wheat but very few reports are available on cytokinin-mediated regulation of yield in dicot plant species (Schwarz et al., 2020a). The First study on cytokinin-mediated regulation of yield through the activity of CKX was reported in the model dicot plant species *A. thaliana.* Double knockout lines (*ckx3ckx5*) of *CKX3* and *CKX5* which are expressed in the organizing center of inflorescence meristem and floral meristem at different growth stages as well as developing gynoecia primordia and placenta tissues, exhibited a stronger stem, enlarged inflorescence meristem having increased number of cells, larger flowers, larger gynoecium having nearly as twice as many ovules as wild type, 40% increase in the number of siliques formed and 55% increase in yield output as compared to wild-type control plants (Bartrina et al., 2011b). *WUSCHEL* encodes a homeodomain transcription factor that positively regulates stem cell population in the shoot apical meristems and floral meristems and thus acts as the master regulator of shoot and floral meristem size and integrity in flowering plants (Jha et al., 2020). RNA *in situ* hybridization revealed that the expression domain of *CKX3* overlaps with *WUSCHEL* expression domain and is significantly enlarged in *ckx3ckx5* indicating that increased endogenous cytokinin might be modulating the size and activity of inflorescence meristem through the *WUSCHEL-CLAVATA3* regulatory loop (Bartrina et al., 2011a). In an excellent example of translational research sextuple *ckx3ckx5* mutant was developed in allotetraploid *B. napus* using random EMS mutagenesis followed by TILLING and backcrossing for four generations. *ckx3ckx5* sextuple mutant exhibited a 52% increase in the number of flowers, 47% increase in the number of pods, 16% increase in the diameter of inflorescence meristem, 24% increase in the number of floral primordia, 32% increase in the number of ovules, 20% increase in total seed weight, increase in the total number of seeds per plant as well as TGW. Although the total seed number was increased as compared to the wild-type controls the number of seeds per pod was equivalent to wild-type plants because of high seed mortality in *ckx3ckx5* mutant (Schwarz et al., 2020a). In both *A. thaliana* and *B. napus* mutations in *ckx3* or *ckx5* alone were not sufficient to alter yield parameters significantly indicating that contrary to their monocot counterparts, simultaneous silencing of more than one *CKX* gene might be required to increase the yield parameters significantly. Recently, three SNPs resulting in non-synonymous substitutions in *GmCKX7-1* which is the most highly expressed *CKX* member during pod and seed developmental stages were found to be associated with high yielding phenotype, and cultivars having these 3 SNPs in *GmCKX7-1* had high seed weight (OAC Wallace, DH420, IA1010LF) and high yield (DH748) (Nguyen et al., 2021b). Among the 3 SNPs identified, H105Q is thought to alter the FAD cofactor binding site critical for enzyme function and thus negatively affects the enzyme’s structural integrity which might result in up to 100-fold lower enzyme activity which in turn will result in increased endogenous cytokinin content during seed development stages effectively increasing the yield output. Indeed among all the cultivars analyzed, the concentration of CK-nucleotide and free base forms during pod and seed development was highest in high-yielding cultivars DH420 and OAC Wallace (Nguyen et al., 2021a). Analysis of Tnt1 retrotransposon tagged mutants in model legume *M. truncatula* revealed that mutant lines having insertion in a single *CKX* gene were similar to wild-type control lines at both vegetative and reproductive stages (Wang et al., 2021b). The only exception was the *ckx3* mutant which exhibited a decrease in primary root length and an increase in the number of lateral roots. These results suggest that there is functional redundancy among different *MtCKX* gene family members in terms of regulation of root-shoot architecture and yield output which further indicate the necessity of downregulating more than one *CKX* gene in dicots to get a distinct detectable phenotype (Wang et al., 2021a).

### 1.4 *CKX* genes as a genetic target for improvement of root-shoot architecture, stress tolerance, and micronutrient acquisition

Root-system architecture (RSA) is primarily responsible for nutrient and water uptake, biotic rhizospheric interactions, and soil anchorage in flowering plants (Lombardi et al., 2021). RSA is quite flexible as root and shoot growth is differentially favored in response to varying environmental conditions (Koevoets et al., 2016). Under optimum environmental conditions having plenty of water and minerals, growth of shoot is promoted and root growth is maintained at a level sufficient to sustain shoot growth while under water and mineral deficient conditions extensive root growth is promoted to trap more water and minerals and shoot growth is restrained to avoid excessive water loss and mineral depletion. This root-shoot architecture is majorly controlled by the antagonistic effects of two phytohormones auxin and cytokinins (Kurepa and Smalle, 2022). Cytokinins directly repress the formation and development of lateral root primordium and negatively regulate the transcription of genes encoding PIN transporter proteins, effectively disrupting PIN-dependent auxin maxima required for lateral root development (Jing and Strader, 2019). The presence of “Casparian strips” and suberin lamellae in the endodermal cell layer negatively regulates apoplastic and transcellular pathways of radial ion transport into the central vascular cylinder (Bao et al., 2019). The endodermis is occasionally interrupted by cells known as “Passage cells” having reduced suberin deposition which leads to a compromised ability to restrict transcellular ion transport (El-Showk and Mähönen, 2018). Auxin-mediated repression of cytokinin signaling was found to impart passage cell fate to endodermal cells adjacent to the xylem pole indicating that cytokinin negatively regulates the passage cell formation and thus also negatively regulates ion uptake through the transcellular pathway (Andersen et al., 2018). Asymmetric cytokinin signaling at the upper part of nascent lateral roots reduces the growth at the upper part and thus acts as an anti-gravitropic signal counteracting against gravity-induced, auxin-dependent cell elongation at the lower root part, ultimately determining the gravitropic set-point angle (GSA) which itself is a key determinant of soil exploration capacity of RSA (Waidmann et al., 2019; Waidmann and Kleine-Vehn, 2020; Lombardi et al., 2021). Cytokinins are also known to negatively regulate nutrient uptake by repressing the expression of micronutrient transporter genes for several minerals such as zinc (Gao et al., 2019), nitrate (Kiba et al., 2011), sulfate (Maruyama-Nakashita et al., 2004), phosphate (Martín et al., 2000) and, iron (Séguéla et al., 2008). Cytokinins also negatively affect plant’s response to water deficiency and drought stress through a plethora of diverse mechanisms including promotion of shoot growth and inhibition of root growth, increased stomata density and transpiration (Farber et al., 2016), direct interference with ABA-mediated stomatal closure responses and repression of ABA-inducible stress-related genes (Salvi et al., 2021; Kurepa and Smalle, 2022). Increased cytokinin signaling in type B *ARR1* gain of function mutant indicated that cytokinins induce the expression of ribosomal protein genes *RPL4A* and *RPL4D* which resulted in an increase in the overall rate of global protein synthesis in *Arabidopsis* culminating in growth inhibition and hypersensitivity to osmotic stress which can be reversed by ABA treatment (Karunadasa et al., 2020). Type B-ARRs bind to promoters of cytokinin responsive genes after getting phosphorylated by Histidine phosphotransfer proteins (AHPs) and activate the transcription of cytokinin responsive genes in response to cytokinin signaling (Kieber and Schaller, 2018b). A recent report reveals the negative role of cytokinins in a plant’s defense response to salinity stress as type B ARR1, 10, and 12 are degraded through MPK3 and MPK6 mediated signaling cascade under salinity stress eventually increasing the salt stress tolerance in *Arabidopsis* (Yan et al., 2021). Triple mutants of genes encoding type-B RR - *arr1,10,12* triple mutant exhibited significant increase in drought tolerance (Nguyen et al., 2016). Defective cytokinin signaling in *Arabidopsis ahp2,3,5* and *arr1,10,12* mutants resulted in increased accumulation of primary metabolites (sugars and amino acids), secondary metabolites such as anthocyanins and flavonoids, and extensive lipid profile reprograming which might have contributed to enhanced salinity stress tolerance of mutant plants compared to wild type plants. Moreover, the accumulation of secondary metabolites, lipids, and sterols was strongly correlated with altered expression of genes pertaining to the biosynthetic pathway of concerned metabolites. These results indicate that cytokinin signaling negatively regulates plant’s defense response to salinity stress through modulating the transcription of specific metabolic pathway genes (Abdelrahman et al., 2021). Similarly, loss of function mutants (*ahp2,3,5*) of genes encoding Histidine phosphotransfer proteins (AHPs) which are positive regulators of cytokinin signaling, resulted in a strong drought-tolerant phenotype (Nishiyama et al., 2013). In perfect alignment with the statements stated above, local or systemic decrease in active cytokinin pool through constitutive or tissue-specific overexpression of *CKX* genes encoding cytokinin degrading cytokinin oxidase/dehydrogenase resulted in improved root-shoot ratio, increased abiotic stress tolerance, and enhanced micronutrient acquisition in several plant species as reviewed below (See Figure 2; Table 3).

**TABLE 3:** Genetic manipulation of *CKX* genes for drought tolerance and enhanced micronutrient acquisition.

S.No.	Name of *CKX* gene	Source species	Target species	Type of genetic manipulation	Observed effects of genetic manipulation	Comments	References
1	*AtCKX1 and AtCKX2*	*Arabidopsis thaliana*	*Nicotiana tabacum*	Constitutive overexpression under *CaMV35S* promoter	Lateral and adventitious roots number ↑	Dwarfed shoot phenotype and size of shoot apical meristem and number of flowers decreased	Werner et al. (2010)
					Length of primary root ↑		
					Root biomass ↑		
2	*AtCKX1 and AtCKX3*	*Arabidopsis thaliana*	*Nicotiana tabacum, Arabidopsis thaliana*	Root specific expression under *WRKY6* and *PYK10* promoters	Lateral and adventitious roots number ↑	No obvious adverse effect on shoot attributes	Werner et al. (2010)
					Length of the primary root ↑		
					Root biomass ↑		
					Root: shoot biomass ratio↑		
					Nutrient stress tolerance ↑		
					Micronutrient content in leaves ↑		
					Phytoremediation capacity ↑		
3	*AtCKX2*	*Arabidopsis thaliana*	*Brassica napus*	Constitutive overexpression under *CaMV35S* promoter	Length of primary root ↑	No obvious adverse effects on shoot attributes	Nehnevajova et al. (2019)
					Lateral root density ↑		
					Adventitious roots number ↑		
					Root biomass ↑		
					Tolerance to nutrient limitation ↑		
					Leaves micronutrient content ↑		
					Phytoremediation capacity ↑		
4	*AtCKX1 and AtCKX2*	*Arabidopsis thaliana*	*Hordeum vulgare*	Root specific expression utilizing *pEPP* promoter	Root length ↑	Shoot growth and development were not compromised in transgenic lines	Ramireddy et al. (2018)
					Root surface area ↑		
					Root biomass ↑		
					Root-shoot biomass ratio ↑		
					Micronutrient content in leaves and seeds ↑		
					Tolerance to drought stress ↑		
5	*MsCKX7*	*Medicago sativa*	*Arabidopsis thaliana*	Constitutive overexpression under *CaMV 35S* promoter	Root length ↑		Li et al. (2018)
					Lateral roots number ↑		
					Root fresh weight ↑		
					Tolerance to salinity stress ↑		
6	*CaCKX6*	*Cicer arietinum*	*Cicer arietinum*	Root specific expression under *WRKY31* promoter	Root length ↑	Stem growth and development were not affected and root nodulation was not compromised in transgenic lines	Khandal et al. (2020)
					Lateral roots numbers ↑		
					Root biomass ↑		
					Seed yield output ↑		
					Mineral content in seeds ↑		
					Tolerance to drought stress ↑		
7	*OsCKX4*	*Oryzae sativa*	*Oryzae sativa*	Root specific expression under *RCc3* promoter	Root growth ↑	Shoot growth and development was not compromised in transgenic lines	Gao et al. (2019)
					Root-shoot biomass ratio ↑		
					Zinc content in the root, shoot, and grain ↑		
					Overall yield output ↑		

^#^ ‘↑’ and ‘↓’ symbols respectively indicate increase and decrease in the corresponding parameter

#### 1.4.1 Model plants

Constitutive overexpression of AtCKX1 and AtCKX2 under CaMV35S promoter in *Nicotiana tabacum* established the opposite role played by cytokinin in root and shoot growth and development. Endogenous cytokinin content was reduced by 31%–63% in transgenic lines which in turn resulted in enhanced root growth marked by the increased size of root apical meristem (RAM), rapid elongation of the primary root, up to 60% increase in root diameter, increased production of lateral and adventitious roots resulting in overall ∼60% increase in dry root biomass. However shoot growth was adversely affected in transgenic plants indicated by a reduction in shoot meristem (SAM) size, dwarfed growth habit, formation of lanceolate epinastic leaves having severely reduced surface area, delayed flowering with a reduction in the number of flowers formed per plant as compared to wild type plants (Werner et al., 2010). Root specific expression of *AtCKX1* in *N. tabacum* using root-specific *WRKY6* promoter validated the better feasibility of tissue-specific expression of *CKX* genes compared to ectopic expression for altering the root-shoot architecture as homozygous transgenic T2 lines exhibited up to a 50% increase in primary root elongation, 27%–39% increase in fresh root biomass and as a consequence root-shoot ratio increased by up to 40% in transgenic lines while the shoot phenotypic parameters (shoot height, number of leaves and time of flowering) were identical to wild type plants. Transgenic lines also exhibited a higher survival rate under drought stress and increased biomineral uptake capacity as indicated by increased accumulation of Zinc (57%), Sulphur (43%), Manganese (33%) and, Phosphorus (46%) in leaves of transgenic lines compared to wild type plants (Werner et al., 2010).

#### 1.4.2 Monocot crop plants

Barley transgenic lines developed through the root-specific expression of *AtCKX1* under mild root-specific promoter *bGLU* derived from maize exhibited enhanced recovery rate and increased shoot biomass under mild and severe drought conditions (Pospíšilová et al., 2016). T₃ transgenic homozygous lines developed by root-specific expression of *AtCKX1* and *AtCKX2* in barley utilizing root-specific *pEPP* promoter system developed larger root systems characterized by a 24%–70% increase in root length, 12%–50% increase in root surface area, 47% increase in root biomass and 16%–50% increase in root-shoot biomass ratio while shoot growth and yield parameters were equivalent to wild type plants. Transgenic lines showed enhanced accumulation of several macro and micronutrients in leaves (13%–53% increase in phosphorus, 17%–45% increase in sulfur, 20%–28% increase in copper, 51%–70% increase in manganese and, 7%–54% increase in zinc) and seeds (increase in calcium, copper and 26%–49% increase in zinc). Enhanced root growth also facilitated improved tolerance to drought stress in transgenic lines indicating 25%–29% stomatal conductance (SC), 30%–32% relative water content (RWC), and 33%–45% CO₂ assimilation rate as compared to 11% SC, 18% RWC and 13% CO₂ assimilation rate in control lines. The endogenous ABA and Proline levels increased by 11 and 620 fold in wild-type plants compared to a corresponding increase of 4–5 folds and 20%–50 fold in transgenic lines (Ramireddy et al., 2018b). ABA is a master regulator of a plant’s defense response against several abiotic stresses (Vishwakarma et al., 2017). Similarly, amino acid proline accumulates under a variety of abiotic stresses such as drought and oxidative stress conditions. Proline works as an excellent osmoprotectant, compatible solute, ROS scavenger and stabilizes membrane integrity and protein structures (Hayat et al., 2012). ABA and proline accumulation are the markers for drought stress. Relatively decreased accumulation of ABA and proline in transgenic lines under drought stress compared to control plants indicate that transgenic lines experience weaker stress levels as detrimental effects of increased ABA content on photosynthesis and growth are minimized (Ramireddy et al., 2018c). Field grown transgenic barley lines validated the data obtained under controlled conditions as they showed an 8%–30% increase in zinc concentration, 16%–32% increase in iron, and 22%–36% increase in manganese concentration compared to wild type plants grown under the same conditions (Ramireddy et al., 2018a). In rice, a dominant gain of function mutant *ren1-D* was identified through enhancer mediated activation tagging having an enlarged root system marked by an increased number of crown roots, increased root length, and increased root dry weight however shoot height was reduced as compared to control plants. Eventually, based on functional genetic analysis the mutant phenotype was attributed to enhanced expression of *OsCKX4* in mutant lines. Root-specific expression of *OsCKX4* under root-specific *RCc3* promoter led to enhanced root growth without any adverse effect on shoot phenotype eventually increasing the root-shoot biomass ratio of transgenic lines (Gao et al., 2014). Recent evidence indicates that crown root growth and development is negatively regulated by the *OsNAC2* transcription factor in rice. *OsNAC2* overexpression lines were marked by increased expression of *OsCKX4* and *OsCKX5* genes and decreased expression of *OsIPT3, OsIPT5,* and *OsLOG3* genes compared to control lines, effectively decreasing the number of crown roots and root length in *OsNAC2* overexpression lines. Moreover, OsNAC2 was shown to directly interact with the *OsCKX4* promoter along with several auxin-related genes establishing itself as a central integrator of auxin-cytokinin crosstalk involved in rice root development (Mao et al., 2020). In Rice, exogenous cytokinin application decreased the Zn-uptake under normal and Zn-limited conditions through downregulation of *OsZIP1* and *OsZIP5* which encode the metal transporters involved in Zn-uptake. Transgenic lines expressing *OsCKX4* under root-specific *RCc3* promoter accumulated 45%–67% more Zn in roots and 60%–68% more Zn in shoots as compared to wild-type control lines under both controlled and field conditions. Moreover, a 57%–61% increase in Zn concentration was reported in brown rice derived from transgenic lines along with an overall 10.9%–11.2% increase in crop yield per plot indicating the presence of cytokinin dependent regulatory module for Zn uptake in rice (Gao et al., 2019).

#### 1.4.3 Dicot crop plants

Transgenic *Brassica rapa* seedlings generated by constitutive overexpression of *AtCKX2* under *CaMV35S* promoter exhibited a 25%–35% increase in primary root length, 70%–100% increase in lateral root density, 40%–50% increase in adventitious roots as compared to wild type lines under *in-vitro* growth conditions as well as up to 50% increase in dry root biomass when grown in soil or hydroponic culture. Moreover, there was no evident growth penalty on shoot development except for the partial release of apical dominance as lateral buds in transgenic lines formed up to 2 small leaves at maturity. Leaves of transgenic plants accumulated 13%–16% more Phosphorus, 41%–56% more calcium, 42%–75% more sulfur, 29%–32% more magnesium, 26%–32% more zinc, 28%–29% more copper, and 15%–20% more manganese while 11%–18% reduction in iron concentration was reported as compared to non-transgenic lines (Nehnevajova et al., 2019). Transgenic chickpea lines generated through the root-specific expression of *CaCKX6* under root-specific *CaWRKY31* promoter formed a larger root system marked by a 1.8-fold increase in lateral root numbers, 1.5–1.85 fold increase in root length, 1.5–2 fold increase in root biomass and 1.7 fold increase in root-shoot biomass ratio. Moreover, transgenic lines showed up to a 20% increase in shoot biomass and a 15%–25% increase in the number of seeds formed per plant. There was no difference in root nodule formation frequency between wild-type and transgenic lines. Owing to higher mineral uptake capacity imparted by larger root systems, transgenic lines accumulated higher amounts of minerals in their seeds as indicated by accumulation of 27–62% more zinc, 26–61% more copper, 22–48% more iron, 13–22% more magnesium, 11–27% more potassium and 5–19% more phosphorus. Transgenic lines also showed improved tolerance to long-term drought stress marked by relatively less decrease in relative water content (RWC), stomatal conductance, transpiration rate, and CO₂ assimilation rate as compared to control line subjected to similar drought conditions (Khandal et al., 2020).

### 1.5 Cytokinin oxidase/dehydrogenase inhibitors—A potential alternative to genetic manipulation of *CKX* genes

Besides molecular biology-based cytokinin oxidase inhibition through genetic manipulation of *CKX* genes, exogenous application of chemical-based cytokinin oxidase inhibitors can also be used to suppress CKX enzyme activity and increase endogenous cytokinin levels (Arora and Sen, 2022) Thidiazuron (*TDZ*), diphenylurea and its derivatives like CPPU, DCPPU were the first cytokinin oxidase inhibitors. However, these exhibit cytokinin activity as well which is usually remarkably higher than CKX inhibitory activities (Kopečný et al., 2010). HETDZ and 3FMTDZ derived from TDZ exhibited significantly lesser intrinsic cytokinin activity and 15-fold lower IC₅₀ values as compared to TDZ for AtCKX2, ZmCKX1, and ZmCKX4a indicating higher CKX inhibiting potential of HETDZ and 3FMTDZ (Nisler et al., 2016). Recently developed CKX inhibitors derived from diphenylurea, CPPU and DCPPU did not exhibit any intrinsic cytokinin activity and one of these novel CKX inhibitors named 1-[2-(2-hydroxyethyl)phenyl]-3-[3-(trifluoromethoxy)phenyl] urea increased seed yield and stress tolerance in *Arabidopsis* as well as increased grain yields in field-grown rapeseed, wheat, and barley crops (Gupta et al., 2021; Nisler et al., 2021). CKX inhibitors have a wide range of applications in horticulture, agriculture, and plant biotechnology as they improve grain yield, enhance abiotic stress tolerance, and improve organogenesis and regeneration in plant tissue culture (Gupta et al., 2021). Chemical CKX inhibitors are attractive potential alternatives for CKX inhibition strategies based on genetic engineering approaches.

## 2 Conclusion and future perspectives

Optimum root-shoot ratio, improved root system architecture, and increased seed yield are desirable traits for crop plants for efficient uptake and utilization of limited soil and water resources but their incorporation into crop plants is rather complicated, laborious, and inefficient because these traits are generally regulated by multiple genes. Genetic manipulation of a single or couple of dominant master regulator genes such as *CKX* genes provides us with an unmatched opportunity to alter these multigenic traits for the development of improved crop cultivars having increased yield and enhanced resilience to adverse environmental fluctuations. Although theoretically feasible, genetic manipulation of *CKX* genes poses several practical challenges because of the activation of a strong homeostatic regulatory mechanism. Activation of homeostatic regulatory mechanisms might prevent the delivery of anticipated results or even necessitate the simultaneous knockdown or knockout of more than one *CKX* gene to get the desirable phenotype as observed in dicot crop plants. Endogenous cytokinin level alteration through genetic manipulation of *CKX* genes is analogous to dealing with a “double-edged” sword since cytokinins regulate root and shoot growth in an opposite and antagonistic manner. Thus, careful selection of target *CKX* genes and choice of tissue-specific transgene expression system is crucial to avoid undesirable pleiotropic effects. *CKX* genes exist as small multigene families in flowering plants, different members of which are more often than not diverged among different species and thus different *CKX* genes play different roles in different species. Genome-wide identification and functional characterization of the *CKX* gene family is an essential prerequisite for the identification of suitable target *CKX* genes in a species-specific manner. Genetic manipulation of *CKX* genes specifically gene knockout and knockdown is a tricky task in polyploid crops as it requires simultaneous knockout or knockdown of all the homologs of a specific *CKX* gene in all the sub-genomes of a polyploid crop. This tremendous feat has been achieved through a set of specific techniques such as random mutagenesis, TILLING, and crossing in allotetraploid oilseed rape. CRISPR/Cas9 mediated gene editing is a versatile method of mutating genes as it can induce mutations at multiple genomic sites simultaneously. Designing the sgRNA from a highly conserved region that targets all the gene copies simultaneously is the most feasible strategy to achieve the knockout of multiple homologs of specific *CKX* genes present in the complex polyploid genomes. Although CRISPR/Cas9 mediated gene editing has been optimized to edit genes in several polyploid crops (as reviewed in Schaart et al., 2021), there are no reports of CRISPR/Cas9 mediated knockout of *CKX* family genes in any polyploid crop species to date. Understanding the precise molecular mechanism behind enhanced micronutrient uptake capacity of *CKX* overexpression lines would help design strategies for the development of bio-fortified crop cultivars enriched in specific nutrients. To date, genetic manipulation of *CKX* genes has been utilized independently for yield enhancement and root growth improvement. Thus, an interesting, future endeavor would be simultaneous upregulation (overexpression) of *CKX* in roots and downregulation (knockdown or knockout) in shoots or grains to develop high yielding, bio-fortified, and stress-tolerant crop cultivars. Another engrossing prospect open for exploration in near future is the simultaneous enhancement of sink strength and source capacity through downregulation of one or more *CKX* genes in shoots or grains and expression of cytokinin biosynthetic *IPT* genes in leaves which might ameliorate the negative effects of complete gene knockdown such as low seed setting rate, high seed mortality and lower TGW. In conclusion, the role of *CKX* genes in determining yield, nutrient uptake, and root-shoot architecture is evolutionary conserved and is of functional importance to most flowering plants thus careful genetic manipulation of specific target *CKX* genes might be the key to developing improved crop cultivars having high yield, improved nutrient status and enhanced tolerance to abiotic stress conditions.
